# Medical Treatment of a Staghorn Calculus: The Ultimate Noninvasive Therapy

**DOI:** 10.1089/cren.2015.29003.jdc

**Published:** 2015-10-01

**Authors:** Joshua D. Chamberlin, Ralph V. Clayman

**Affiliations:** Department of Urology, University of California, Irvine, Orange, California.

## Abstract

A 77-year-old female presented with bilateral staghorn calculi. She underwent an uneventful left percutaneous nephrolithotomy (PCNL); the stone analysis revealed a 90% struvite and 10% calcium phosphate stone. Treatment of the right stone was postponed by the patient. During the next 9 months, her family physician gave her multiple courses of culture-directed antibiotics due to breakthrough urinary-tract infections, despite her also being on a prophylactic antibiotic. After 9 months, she agreed to undergo her right PCNL. Preoperatively, a non-contrast CT scan was obtained; it revealed complete resolution of the right staghorn calculi.

## Clinical History

A 77-year-old female was referred due to a history of intermittent left flank pain and recurrent urinary-tract infections. Laboratory studies by her physician revealed worsening renal function; subsequently, a CT scan showed bilateral >3-cm renal staghorn calculi involving three renal calyces bilaterally with moderate left hydronephrosis. Hounsfield units on the left stone were 694 and on the right stone were 664. Significant in her medical history was a parathyroidectomy 7 years ago for hyperparathyroidism, discovered following metabolic workup for nephrolithiasis; serum parathyroid hormone and calcium levels returned to normal. Her medical history included hypertension, chronic kidney disease, arthritis, atrial fibrillation, hepatitis B, hyperlipidemia, and anemia. Her prior surgeries included bilateral hip and knee prostheses and oophorectomy. Her medications included prophylactic dose cephalexin, lisinopril, furosemide, valsartan, calcium carbonate, acetaminophen with codeine, colace, amiodarone, warfarin, lovastatin, and iron. Her family history revealed kidney stones in her daughter.

She underwent an MAG-3 renal Lasix scan, which showed split function of 49.3% on the left and 50.7% on the right, with decreased drainage on the left side (T½ of 22.17 minutes on the left *vs* 5.33 minutes on the right) with associated left hydronephrosis. After a 7-day course of levofloxacin, she underwent an upper pole left percutaneous nephrolithotomy (PCNL) with bridging anticoagulation therapy. A low dose CT scan of the abdomen and pelvis without contrast the following morning revealed resolution of the left collecting system staghorn with persistence of a 4 mm and 8 mm upper and lower pole renal parenchymal calcification, respectively; the right staghorn calculus was unchanged. Stone analysis demonstrated 90% struvite and 10% calcium phosphate, with stone culture positive for *Enterococcus faecalis* and *Proteus mirabilis*. She was discharged from the hospital on postoperative day 3. She was scheduled to undergo right PCNL 6 weeks later, but postponed her surgery due to her husband's poor health and ultimate passing. As such, her surgery was delayed for 9 months.

## Physical Examination

Examination revealed an elderly Caucasian female who was 5.0 feet tall with body mass index of 32.5 kg/m^2^. Her blood pressure was 167/67 with normal remaining vital signs. She had a 2/6 systolic ejection murmur. Abdomen was soft with a well-healed midline abdominal incision. There was no costovertebral tenderness bilaterally. PCNL tract on the left showed a well-healed scar. There was 1+ peripheral edema. The remainder of the examination was unremarkable.

*Diagnostic studies urinalysis:* Specific gravity 1.012, pH 7.5, protein 100, nitrite positive, leukocyte esterase positive, red blood cell count >182, white blood cell count 176. Urine culture: >100,000 CFU of *E. faecalis* and 11,000 CFU of *P. mirabilis*.

*Laboratory studies:* White blood cells count 7.1, hemoglobin 12.7, hematocrit 37.2, platelets 206. International normalized ratio 2.5, sodium 140, potassium 4.4, chloride 104, carbon dioxide 23, blood urea nitrate 53, creatinine 2.2, glomerular filtration rate 31, glucose 85, calcium 8.8, magnesium 1.8, phosphorous 3.2.

**FIG. 1.**
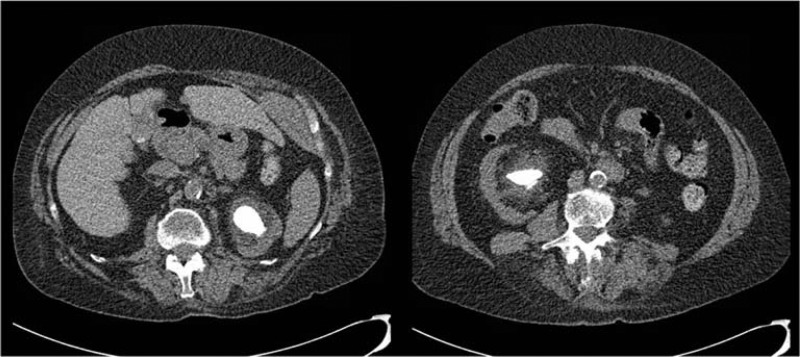
CT scan prior to left PCNL: Bilateral staghorn stones, left 3.2 cm renal staghorn involving 3 calyces, HU of 694, and right 3.4 cm renal staghorn involving 3 calyces, HU of 664. There is mild left hydronephrosis, bilateral parenchymal thinning and two left renal parenchymal calcifications (not shown).

*Initial CT scan prior to left PCNL (Fig. 1):* A left 3.2 cm renal staghorn involving three calyces with HU of 694 and a right 3.4 cm renal staghorn involving three calyces with HU of 664. There are two separate left renal parenchymal calcifications, 4 mm and 8 mm, left mild hydronephrosis, and bilateral parenchymal thinning.

*CT scan 1 day following left PCNL:* Complete resolution of left staghorn stone with persistence of 4 mm and 8 mm left renal parenchymal calcifications and persistence of a 3.4-cm right renal staghorn.

**FIG. 2.**
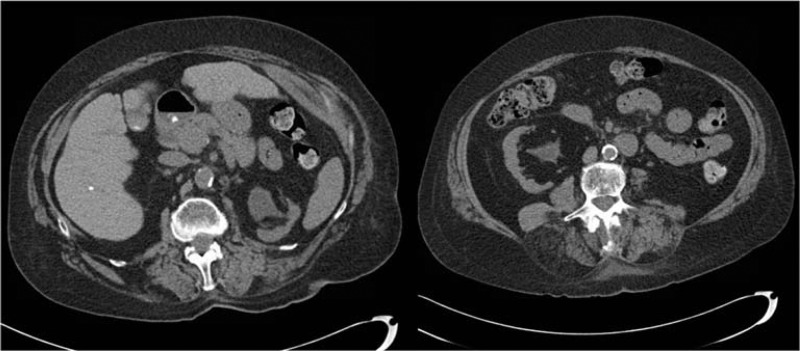
CT scan after 9 months of antibiotic therapy: No new left renal stones, improvement of left hydronephrosis and complete resolution of right renal stone.

*CT scan 9 months later, prior to scheduled right PCNL (Fig. 2):* Unchanged left renal parenchymal calcifications, no new left renal stones, improvement of left hydronephrosis and complete resolution of right renal stone.

## Intervention

She was scheduled to undergo PCNL on the right, but the patient delayed surgery due to her husband's ill health and subsequent death. During this time, she was placed on a 5-month course of prophylactic antibiotics cephalexin 250 mg PO daily and a 1-month prophylactic course of nitrofurantoin 100 mg PO daily, but continued to have symptomatic urinary-tract infections that were positive for *E. faecalis*, *P. mirabilis*, *Escherichia coli*, and *Klebsiella pneumonia* (Table 1). As such, over the next 9 months, she underwent 17 culture-directed therapeutic courses of antibiotics prescribed by her family physician, often with two antibiotics given concomitantly. The antibiotics included nitrofurantoin, ciprofloxacin, levofloxacin, sulfamethoxazole–trimethoprim (SMX-TMP), and amoxicillin (Table 2).

**Table 1.** Urine Cultures

*Urine source*	*Bacteria*	*Amount (CFU)*	*Susceptible*	*Resistant*
Clean midstream	*Enterococcus faecalis*	>100,000	Nitrofurantoin, ampicillin	Ciprofloxacin
	*Proteus mirabilis*	11,000	SMX-TMP, ciprofloxacin, cephalexin	Nitrofurantoin
Cystoscopy	*E. faecalis*	>100,000	Nitrofurantoin, ampicillin	Ciprofloxacin
Left renal stone	*E. faecalis*	2+	Nitrofurantoin, ampicillin	Ciprofloxacin
	*P. mirabilis*	Few	SMX-TMP, ciprofloxacin, cephalexin	Nitrofurantoin
Clean midstream	*E. faecalis*	>100,000	Nitrofurantoin, ampicillin	Ciprofloxacin
	*Escherichia coli*	4000	Nitrofurantoin, SMX-TMP, cephalexin	Ciprofloxacin
Clean midstream	*E. faecalis*	>100,000	Nitrofurantoin, ampicillin	Ciprofloxacin
	*Klebsiella pneumonia*	1000	Nitrofurantoin, SMX-TMP, cephalexin, ciprofloxacin	
	*E. coli*	1000	Nitrofurantoin, SMX-TMP, cephalexin, ciprofloxacin	
Clean midstream	*E. faecalis*	>100,000	Nitrofurantoin, ampicillin	Ciprofloxacin
	*E. coli*	1000	Nitrofurantoin, ciprofloxacin	SMX-TMP, cephalexin

SMX-TMP, sulfamethoxazole–trimethoprim.

**Table 2.** Antibiotics

	*Dose*	*Duration*	*No. of treatments*
Prophylactic antibiotics			
Cephalexin	250 mg daily	5 months	
Nitrofurantoin	100 mg daily	1 month	
Therapeutic antibiotics			
Nitrofurantoin	50–100 mg BID	7–14 days	6
Ciprofloxacin	500 mg BID	7 days	3
Levofloxacin	250 mg daily	7 days	2
SMX-TMP	800 mg/160 mg BID	7 days	4
Amoxicillin	500 mg BID	7 days	2

## Outcome

The patient was rendered stone free of renal collecting system stones on the left following the left PCNL, with only a 4 mm and an 8 mm calcification persisting in the renal parenchyma. The patient was found to have complete dissolution of the right renal stone after 9 months of myriad antibiotic therapies.

According to the 2005 AUA guidelines on the management of staghorn calculi, standard of care includes definitive treatment of newly diagnosed otherwise healthy patients with staghorn calculi to render them stone free with intervening procedures.^1^ Several studies have shown that nonsurgical management of staghorn calculi with antibiotics, urease inhibitors or supportive measures lead to renal deterioration, recurrent urinary-tract infections, sepsis, pain, and increased mortality.^2^

While antibiotics alone have been shown to be insufficient in the definitive management of struvite stones, they play a clear role for the safe management of planned directed therapy. Both the EUA and AUA recommend antibiotic therapy in the presence of a suspected struvite stones with associated infection; however, while antibiotics are typically given for 1 to 2 weeks prior to the planned surgical procedure, high-level guidelines for specific antibiotic choice, timing, and duration have not been established.^1,3^ The curious finding of this case is that long-term very aggressive antibiotic therapy in and of itself resulted in complete resolution of a 3-cm presumably struvite stone. This observation may warrant further investigation as to the potential pharmaceutical dissolution of struvite calculi and may warrant obtaining a CT scan prior to PCNL in cases of suspected struvite stone in patients who have had a prolonged course of antibiotic therapy prior to their planned date of surgery.

## Abbreviations Used

CT: computed tomography
HU: Hounsfield units
PCNL: percutaneous nephrolithotomy
SMX-TMP: sulfamethoxazole–trimethoprim
